# DiPTH‐Cystine and PTH‐Cysteine in Disulfide Bond Analysis Using Automated Edman Degradation

**DOI:** 10.1002/psc.70053

**Published:** 2025-08-29

**Authors:** Toni Kühl, Yomnah Y. Elsayed, Alexander Terekhov, Diana Imhof

**Affiliations:** ^1^ Pharmaceutical Biochemistry and Bioanalytics, Pharmaceutical Institute University of Bonn Bonn Germany; ^2^ Department of Pharmaceutical Analytical Chemistry, Faculty of Pharmacy Ain Shams University Cairo Egypt

**Keywords:** conotoxin, cysteine, disulfide bridges, Edman degradation, insulin, peptides

## Abstract

The annotation of disulfide bridges in peptides and proteins can be an elaborate process and requires careful revision of multiple data sets to avoid wrong assignment in the structural analysis. Herein, we provide additional support to elucidate the cysteine connectivity by re‐implementation of Edman sequencing for the analysis of this specific structural feature. By synthesizing diPTH‐cystine and PTH‐cysteine for comparison, we were able to identify the respective derivative during Edman sequencing when a disulfide bond is detected in a peptide. Application of Edman sequencing to selected peptides with two or three disulfide bridges provides further insight into the differentiation of cysteines that form a disulfide bridge for both half‐cystines in the same cycle and in separated cycles. A combined approach for the implementation of automated Edman sequencing in the process of disulfide bond assignment is described to alleviate structural elucidation in the future analysis of cysteine‐rich peptides and proteins.

## Graphical abstract

The mystery of PTH‐cysteine and diPTH‐cystine in the analysis of disulfide bonds is solved. Both derivatives were synthesized and applied to Edman sequencing. A time‐efficient combined workflow applying Edman and MS/MS sequencing for a comprehensive analysis of the complex disulfide connectivity in peptides and proteins was developed.

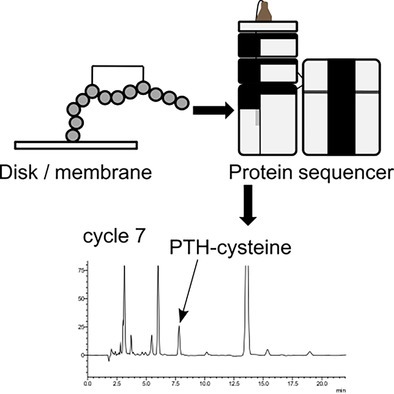

**Abbreviations**
ATZ: anilinothiazolinone
DMPTU: (2,6‐dimethylphenyl)thiourea
DPTU: diphenylthiourea
DTT: dithiothreitol
ED: edman degradation
GFD: glass fiber disk
HPLC: high performance liquid chromatography
LC‐ESI: liquid chromatography‐electrospray ionization
MCP‐1: Macrophage chemoattractant protein‐1
MS: mass spectrometry
NMR: nuclear magnetic resonance
PTH: phenylthiohydantoin
PTC: phenylthiocarbamoyl
PVDF: poly (vinylidene) difluoride
Rf: retardation factor
TCEP: Tris(2‐carboxyethyl)phosphine
TLC: thin‐layer chromatography

## 1 Introduction

The analysis of multiple disulfide bridges in peptides and proteins poses enormous challenges due to the complexity of the three‐dimensional conformations that are formed upon specific linkage of the cysteines [1, 2]. The determination of the disulfide connectivity is crucial for understanding the structural and functional properties of biologically important peptides and proteins [3, 4]. However, this process is often laborious and requires sophisticated techniques such as MS/MS, NMR, or X‐ray analysis, experienced personnel, and a significant amount of time [1, 2, 5, 6]. A recently established protocol for partial reduction combined with automated Edman sequencing and user‐friendly data analysis has simplified the process of identifying the correct disulfide pattern [6, 7]. Still, the sample preparation remains demanding, which highlights the considerable time required to achieve accurate results [1, 6, 7].

A potential approach suggested to accelerate the elucidation of disulfide connectivities in peptides and proteins is based on the observation of diPTH‐cystine (also described as PTH‐bis (cystine), bis (PTH)‐cystine, or PTH‐cystine) formation during Edman sequencing, which was already exemplified for various examples, including human macrophage chemoattractant protein‐1 (MCP‐1), human von Willebrand factor, human plasma kallikrein, human coagulation factor XI, huwentoxin‐II, human beta‐defensins‐1–4, murine interleukin‐6, human complement C1s, and conotoxin GS, in the past [8, 9, 10, 11, 12, 13, 14, 15, 16]. The analysis of the formed diPTH‐cystine for these proteins and fragments thereof containing crosslinked cysteines has been proposed and applied as a method for determining the disulfide connectivity [8, 9, 10, 11, 12, 13, 14, 15, 16]. The results of some of these earlier reports, however, remain ambiguous as, e.g., in the case of MCP‐1, the data presented also suggested that disulfide shuffling may have occurred during the process of fragment preparation [8]. In addition, in several examples potential reduction of diPTH‐cystine in the protocol for Edman degradation was not considered or mentioned at all [8, 9, 10, 11, 12, 14, 15, 16, 17, 18, 19, 20, 21]. Similarly, sufficient proof for diPTH‐cystine being formed if analyzed for comparison could not be given or was not further detailed [8, 21, 22, 23]. In fact, and to the best of our knowledge, only Haniu et al. analyzed the formed product in more detail using mass spectrometry after performing Edman degradation of cysteine; however, they could not identify the exact mass of diPTH‐cystine [22]. Instead, the authors assigned differences in molar masses to the formation of the dehydro‐form of diPTH‐cystine [22]. As Edman sequencing is usually performed using dithiothreitol (DTT) in several reagents, its use should be avoided to form and preserve diPTH‐cystine throughout the reactions of an Edman cycle [13, 24, 25]. Still, sufficient yields and reliable results were primarily obtained when diPTH‐cystine occurred in early cycles and were optimal when the half‐cystines were measured in the same cycle [22, 25, 26, 27]. In cases in which half‐cystines were located at different positions in a sequencing run, the first cycle of a half‐cystine usually did not show any PTH‐amino acid derivative, and only at the cycle of the corresponding second half‐cystine could the diPTH‐cystine derivative or related degradation products be detected [21, 23, 26, 28]. More specifically, the most common degradation product of diPTH‐cystine was dehydroalanine and/or its DTT adduct, which frequently showed up in the respective cycles and especially in increased concentrations in later cycles [9, 24, 25]. The appearance of PTH‐cysteine, and not diPTH‐cystine, as the actual derivative detected primarily in early cycles under reducing conditions of DTT was emphasized in only a very limited number of studies [24, 28]. Nonetheless, annotation of the observed peaks was mostly as diPTH‐cystine, although it was chemically contradicting to the expected product when DTT is present [8, 9, 10, 11, 12, 14, 15, 16, 17, 18, 19, 20, 21, 22, 23, 26, 27]. In fact, Nokihara et al. collected the formed PTH‐derivative and confirmed by fast atom bombardment mass spectrometry the presence of PTH‐cysteine instead of diPTH‐cystine in the cycle of the second half‐cystine [28].

Thus, questions about the reliability and quantifiability of diPTH‐cystine and PTH‐cysteine formation as valuable tools in automated Edman sequencing are still to be explored [23, 25]. Also, the questions whether this method can be utilized effectively in routine sequence analysis or whether its robustness is adequate for different peptide and protein sequences need to be clarified. Ultimately, the transferability of the protocol and method to the only currently available analytical systems, the PPSQ‐51A and the PPSQ‐53A Protein Sequencer (both Shimadzu) remains elusive, too. The Application News No. B111 from Shimadzu highlights the potential of their instruments for identifying diPTH‐cystine in the context of disulfide bond analysis in oxytocin [23]. Specifically, it is supposed to demonstrate (i) the ability to achieve sufficient resolution of the diPTH‐cystine peak in a chromatogram containing all other proteinogenic PTH‐amino acids of the standard mixture and (ii) that the application of high concentrations of the one‐disulfide‐bonded nonapeptide oxytocin (100 pmol) resulted in the formation of only minimal amounts of diPTH‐cystine in the cycle for the second half‐cystine [23]. Since the origin and production of diPTH‐cystine or PTH‐cysteine are not given in this report, the reliability and significance of the correct analysis of the disulfide bridge in the given example are still uncertain as a real standard has not been provided. Thus, one of the key concerns remains the need for reliable and thoroughly tested standard compounds to ensure accurate system calibration, which has, to the best of our knowledge, yet to be addressed for the instruments available in the market. A second aspect to be considered represents the scope of applicability and interpretation of the occurrence of diPTH‐cystine and/or PTH‐cysteine, in particular, their utilization to multiple disulfide‐bonded peptides and proteins [25]. They were considered valuable tools for the identification of disulfide bridges in complex, multiple disulfide‐containing peptides and proteins when Edman sequencing was still popular in the late 80s up to early 2000s, although proof for the existence of diPTH‐cystine was never provided [8, 9, 10, 11, 12, 13, 14, 15, 16, 17, 18, 19, 20, 21, 22, 23, 24, 25, 26, 27, 28]. It is thus appealing to establish appropriate reference compounds to satisfyingly solve an unanswered question after almost half a century.

Apart from the aforementioned drawbacks, the more frequently described methods often require partial reduction and derivatization of the peptide or protein to facilitate disulfide bridge analysis. However, diPTH‐cystine or PTH‐cysteine determination may allow for a more direct and rapid assessment [2, 6, 25]. To figure out if this method could outperform existing techniques, further research is required to identify the specific conditions in which diPTH‐cystine formation occurs and can be reliably and efficiently used for the analysis of different peptides and proteins. In this study, we present a synthesis route for the production of diPTH‐cystine and PTH‐cysteine and discuss their application as standard compounds for the analysis of multiple disulfide‐containing peptides such as insulin, tridegin, and μ‐KIIIA. The establishment of a less complex and faster approach for the elucidation of disulfide connectivities in peptides and proteins by applying automated Edman degradation compared to more elaborate techniques such as tandem mass spectrometry, NMR spectroscopy, or X‐ray crystallography will be discussed.

## 2 Materials and Methods

Solvents of analytical grade (pyridine, triethylamine, diethylether, ethylacetate, chloroform), hydrochloric acid (1 M), trifluoroacetic acid (spectroscopic grade), acetic acid, and acetonitrile (HPLC grade) were obtained from VWR International GmbH (Darmstadt, Germany). Methanol was from Fisher Scientific GmbH (Schwerte, Germany). Phenyl isothiocyanate (8.36 M, 99%), Sequa‐brene, and *n*‐hexane were from Merck KGaA (Darmstadt, Germany); Tris(2‐carboxyethyl)phosphine (TCEP) was from Applichem (Darmstadt, Germany). L‐Cystine was obtained from Reanal (Budapest, Hungary). Reagents for the protein sequencer (phenyl isothiocyanate, trifluoroacetic acid, triethylamine, 37% acetonitrile, ethylacetate and chlorobutane), TFA‐treated glass fiber disks (GFD), poly(vinylidene difluoride) membranes (PVDF) for sample application, and the PTH‐amino acid standard were provided by FUJIFILM Wako Pure Chemical Europe GmbH (Neuss, Germany) or FUJIFILM Wako Pure Chemical Corporation (Osaka, Japan).

### 2.1 Synthesis of DiPTH‐Cystine

L‐Cystine (~0.01 mmol/mL solvent) was dissolved in water. Triethylamine (4 equiv.), methanol, and phenylisothiocyanate (PITC, 4 equiv.) were added under stirring. The reaction mixture was left for 4–5 h under argon, dried in vacuo, and redissolved in 50% ethyl acetate/water. The phenylthiocarbamoyl (PTC) intermediate was extracted into the aqueous phase, which was then acidified to pH 1 with 1 M hydrochloric acid and stirred under argon for 16 h. The reaction mixture was dried in vacuo to obtain the crude product (white powder). The amount of the crude product obtained before work‐up with aqueous extraction was 95%; the final yield after purification was 31%.

### 2.2 Characterization of DiPTH‐Cystine

The crude diPTH‐cystine derivative was purified by HPLC on a Shimadzu LC10 system equipped with a C18 column (Knauer Eurospher 100–5, 250 x 32 mm, 5 μm particle size, 100 Å pore size). Gradient elution was performed using two solvent systems: 0.1% TFA in water (eluent A) and 0.1% TFA in acetonitrile (eluent B). The compounds were separated using an elution gradient of 20–60% eluent B in 40 min at a flow rate of 1 mL/min at 25 °C, and detection was performed at 220 nm. Injection volumes were between 50 and 400 μL. After collection, fractions were freeze‐dried and tested for purity by reinjection in the same HPLC system under the same conditions. The derivative was stored at −20 °C. For identity confirmation, mass spectrometry (LC MS‐ESI), ^1^H‐, and ^13^C‐NMR spectroscopy were performed. For mass spectrometric analysis, an LC‐ESI micrOTOF‐Q III system (Bruker Daltonics GmbH, Bremen, Germany) coupled with a Dionex Ultimate 3000 (Thermo Scientific, Dreieich, Germany) was utilized. Data was analyzed with the Bruker Compass Data Analysis 4.1. For NMR measurements, the diPTH‐cystine derivative was analyzed on a Bruker Avance III HD Ascend 700 MHz spectrometer (Bruker Daltonics GmbH, Ettlingen, Germany). Thin‐layer chromatography (TLC) analysis was accomplished with the following systems: (I) methanol/water (2:1) and (II) acetonitrile/water (2:1) on F254S RP‐18‐coated silica gel glass plates from Merck KGaA (Darmstadt, Germany). Detection was under UV light and by staining using a 0.5% ninhydrin solution in acetone where relevant. Analytical data for diPTH‐cystine were as follows: A retention time of 28.2 min was obtained. Mass was confirmed by observation of a peak at m/z 475.04 (detected as [M + H]^+^, calculated molecular weight: 474.03 g/mol). R_f_‐values for TLC were 0.55 (system I) and 0.57 (system II). For NMR, the following resonances were measured: ^1^H NMR (700 MHz, CDCl3): δ = 1.23 (m), 1.64 (s), 2.00 (m, 1H), 3.41 (m, 1H), 4.66 (ddd, 1H), 7.30 (m, 2H), 7.51 (m, 2H), 8.06 (s, 1H), while ^13^C NMR (700 MHz, CDCl_3_): δ = 41.68, 59.35, 128.59, 129.14, 132.69, 132.73, 172.59, 184.23, indicating the conversion of the diATZ‐cystine into a stable diPTH‐cystine derivative.

### 2.3 Synthesis and Characterization of PTH‐Cysteine

Reduction of diPTH‐cystine to form PTH‐cysteine was performed using 10 mM Tris(2‐carboxyethyl)phosphine (TCEP) in citrate buffer pH 3.0 for 10 min. Chromatographic separation was executed on the LC10 system mentioned before using a gradient of 10–50% eluent B in 40 min at a flow rate of 1 mL/min at 25 °C with detection at 220 nm. Injection volumes were between 50 and 400 μL. More than 95% conversion of diPTH‐cystine into PTH‐cysteine was observed. The purified PTH‐cysteine had a retention time of 22.2 min. Identity was confirmed by mass spectrometry in which a peak at m/z 239.03 (detected as [M + H]^+^, calculated molecular weight: 238.02 g/mol) was obtained. Rf‐values for TLC were 0.58 (system I) and 0.65 (system II). Throughout all experiments, PTH‐cysteine was always applied or stored in 32% acetonitrile containing 0.1% TFA.

### 2.4 Automated Edman Sequencing

An automated protein sequencer PPSQ‐53A (Shimadzu) was applied for N‐terminal sequencing using a Wakosil PTH‐II 4.6 × 250 mm (S‐PSQ) column for isocratic elution at 40 °C (column oven). The mobile phase (40% acetonitrile, 0.1% acetic acid, FUJIFILM Wako Pure Chemical Corporation, Osaka, Japan) was transported at a flow rate of 1 mL/min. Detection of PTH‐amino acid derivatives was at 269 nm. For Edman degradation, diPTH‐cystine (in 48% acetonitrile, 0.1% TFA) and PTH‐cysteine (in 32% acetonitrile, 0.1% TFA) were prepared. The standard mixture of the 19 proteinogenic PTH‐amino acids and PTH‐delta‐threonine was dissolved and diluted with 37% acetonitrile to obtain a final concentration of 25 pmol/50 μL for each amino acid [29]. Of each solution (standard, diPTH‐cystine, PTH‐cysteine) 50 μL were automatically injected directly in the PPSQ‐53A protein sequencer (Shimadzu). Stock and working solutions were stored at −20 °C. For peptide analysis, the samples were dissolved in 37% acetonitrile (or an appropriate solvent) and subsequently applied to a TFA‐treated glass fiber disk (GFD) (previously treated with Sequa‐brene) or PVDF membrane for larger proteins. After drying under a nitrogen stream, sequencing was performed using the standard chemicals recommended for sequencing by Shimadzu (i.e., reducing conditions utilizing DTT were applied). The GFDs were pre‐treated with Sequa‐brene according to the manufacturer's instructions for the application of shorter peptides. By the end of each sequencing cycle, the eluted PTH‐amino acid derivatives were identified by comparison with either the PTH‐amino acid standard mixture, the diPTH‐cystine, or the PTH‐cysteine derivative as a reference compound.

## 3 Results and Discussion

### 3.1 Synthesis of DiPTH‐Cystine

From the repertoire of the 20 proteinogenic amino acids, those with –OH or –SH group on the β‐carbon were already shown by Pehr Edman to be difficult to constitute due to β‐elimination leading to the respective dehydroalanine (for Cys and Ser) or dehydro‐alpha‐aminoisobutyric acid derivatives (for Thr) [30, 31]. Meanwhile, threonine and serine could be obtained, but cysteine still degrades easily under the conditions for Edman sequencing [32]. As such, diPTH‐cystine was applied as a potential tool for the detection of cysteine involved in disulfide bonds [9, 10, 11, 12, 14, 15, 16, 17, 18, 19, 20]. Numerous reports utilized the occurrence of a peak potentially representing diPTH‐cystine [9, 10, 11, 12, 14, 15, 16, 17, 18, 19, 20], while only a few examples are published in which the compound was produced by application of cystine in the sequencing reaction to obtain a standard for application in the analyses [8, 21, 22, 23]. Interestingly, no specific proof was given in any of these studies for the existence of the isolated compound by mass spectrometry, elemental analysis, or NMR spectroscopy. In several cases, there were no specifications given regarding whether Edman sequencing was performed under reducing (involvement of DTT) or non‐reducing conditions [12, 14, 17, 18, 19, 20]. As such, it remained unclarified whether the detected compound was diPTH‐cystine or PTH‐cysteine. Especially, the analyses by Nokihara et al. and Brune et al., when DTT was added to the conversion reaction for the formation of the PTH‐amino acid, highlighted the synthesis of PTH‐cysteine instead of diPTH‐cystine [24, 28]. Nowadays, standard chemicals for this reaction applied in the PPSQ sequencer series by Shimadzu contain DTT in the relevant reagents, but proof for the formation of diPTH‐cystine and/or PTH‐cysteine is still insufficient, although it would be beneficial to support the detection of disulfide bridges as performed earlier [9, 10, 11, 12, 13, 14, 15, 16, 17, 18, 19, 20, 21, 22, 23, 24, 25, 26, 27, 28]. In addition, a suitable workflow and its applicability for the sequencing of disulfide bridges, as well as the relevance of the formation of diPTH‐cystine and/or PTH‐cysteine, were not given in the respective application news [23].

**SCHEME 1:**
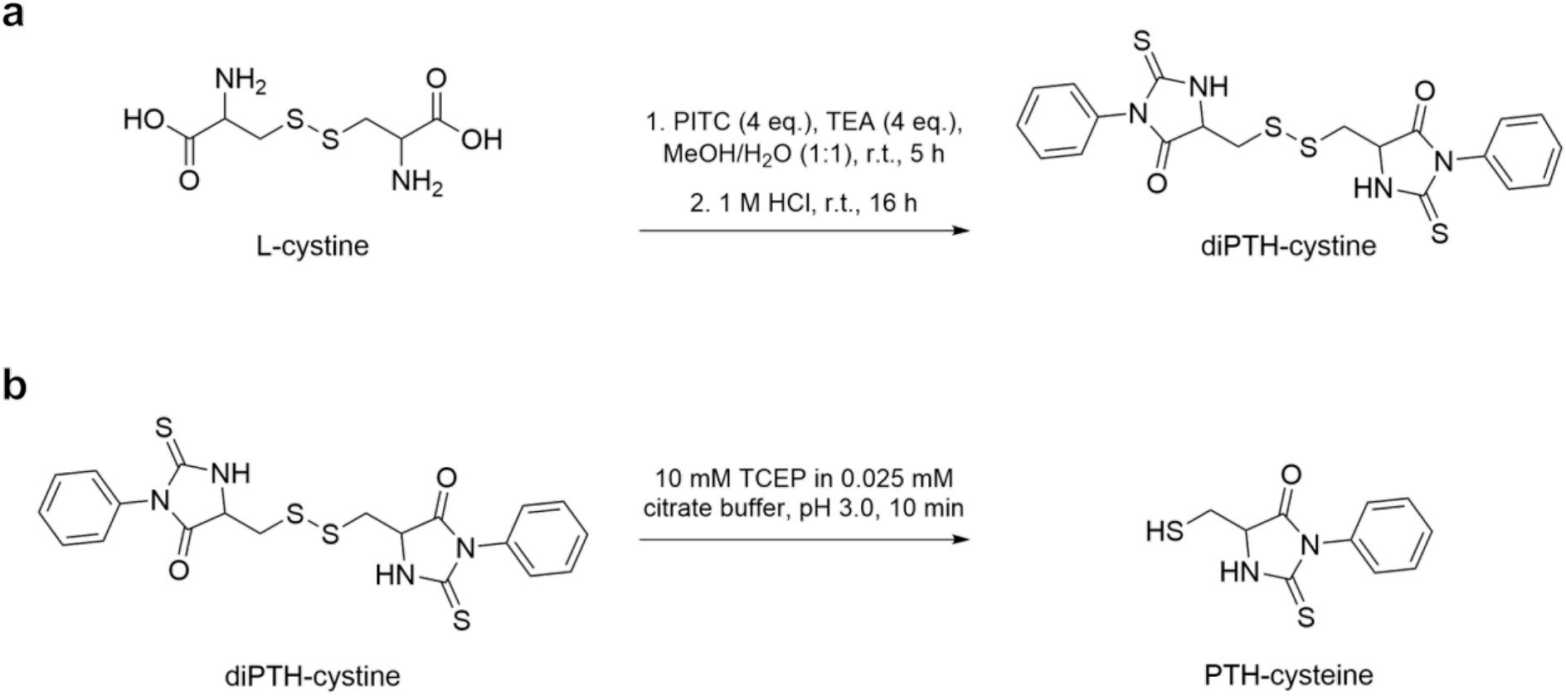
Synthesis scheme for the generation of (a) diPTH‐cystine and (b) PTH‐cysteine.

To identify and confirm the formed compounds in the sequencing runs of the PPSQ sequencer, synthesis of diPTH‐cystine was performed starting from L‐cystine (Scheme 1a). While the addition of PITC was carried out in a green solvent, i.e., a 50% methanol/water mixture, avoiding the typically applied, but more harmful, pyridine/water mixtures to form the PTC‐derivative, the subsequent reactions (cyclization to the ATZ‐derivative and conversion to the PTH‐derivative) were executed according to the standard protocols described earlier [6, 33]. The formation of diPTH‐cystine in sufficient purity and amount could be confirmed accordingly (Supporting Figure S1a, see also Materials and Methods). Reduction of diPTH‐cystine was conducted by the addition of TCEP instead of DTT to avoid the formation of unwanted DTT adducts (Scheme 1b). Complete conversion was quickly obtained; the product was purified, and identity confirmed by mass spectrometry, as shown earlier by Nokihara et al. (Supporting Figure S1) [28].

### 3.2 Incorporation of DiPTH‐Cystine in Edman Degradation

Although identification of diPTH‐cystine was never completely assured during Edman sequencing, reinjections of potential PTH‐derivatives of L‐cystine produced in the sequencer or direct measurement of the compound indicated primarily one peak [8, 21, 23]. However, in longer sequencing runs, the DTT adduct of dehydroalanine was also reported due to degradation of the diPTH‐cystine [28]. In other cases, the occurrence of two peaks was attributed primarily to reduction of the disulfide bond of diPTH‐cystine to form PTH‐cysteine [24, 28]. Nevertheless, appropriate resolution for diPTH‐cystine and its degradation products among each other was always given [9, 12, 24]. However, an overlap with PTH‐derivatives and corresponding degradation products from other amino acids such as PTH‐tyrosine (for diPTH‐cystine) or PTH‐serine (PTH‐dehydroalanine) was observed and required proper analysis to identify the correct amino acid [8, 9, 24]. Brune et al. even suggested performing two sequencing runs for the analysis of disulfide bonds, in which one analysis was performed with DTT added to the reagents, while the second measurement was executed without DTT [24].

As none of the previous devices is available in the market anymore, a careful investigation of the analytical behavior was attempted by Kuriki (Shimadzu) for the PPSQ sequencer series [23]. The authors performed an analysis of diPTH‐cystine and PTH‐cysteine applying cysteine and cystine as samples on polybrene‐treated glass fiber disks and executing the Edman sequencing reaction [23]. Interestingly, in standard isocratic measurements for each compound, two well‐resolved peaks were detected with different ratios at retention times close to PTH‐arginine [23]. They annotated them as PTH‐cysteine and PTH‐cystine according to the different ratios in which they were formed in the corresponding sequencing run [23]. While PTH‐cysteine was also sufficiently well resolved from PTH‐arginine, an overlay of diPTH‐cystine and PTH‐arginine was observed [23]. Unfortunately, no further details on reducing or non‐reducing conditions during their analyses were given.

**FIGURE 1:**
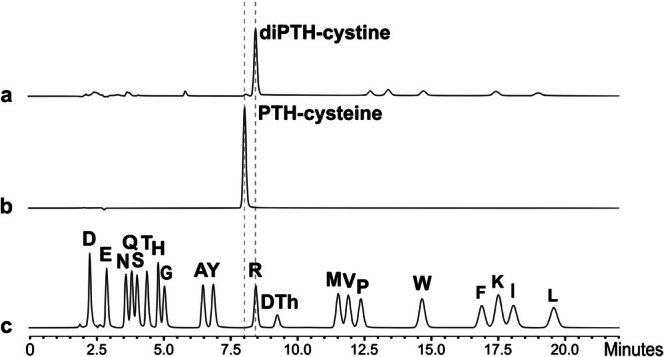
Direct Edman degradation runs of (a) diPTH‐cystine, (b) PTH‐cysteine, and (c) PTH‐amino acids standard mixture in 37% acetonitrile [29].

In terms of the establishment of a workflow for the analysis of disulfide‐containing peptides and proteins, a clear annotation would be advantageous for future analysis of their cysteine connectivity by Edman sequencing. Consequently, our synthesized diPTH‐cystine and PTH‐cysteine derivatives were directly injected as reference compounds for comparison with peptide and protein samples (Figure 1). Upon direct injection, only peaks for the respective pure PTH‐derivative could be observed. For diPTH‐cystine, a retention time of 8.47 min was obtained (Figure 1a), which is to some extent overlapping with the peak for PTH‐arginine (8.56 min, Figure 1c) and thus poorly resolved. In contrast, PTH‐cysteine showed a distinct peak at 8.06 min (Figure 1b) and was significantly different from all other PTH‐derivatives of the standard mixture. These retention times match the annotations performed in the Applications News No. B111 and confirm their results [23]. Interestingly, both compounds did not show any significant degradation during the analytical run; however, it should be considered that application of the reagents applied during Edman sequencing, of course, may influence their stability.

### 3.3 Application of DiPTH‐Cystine and PTH‐Cysteine for Annotation of Multiple Disulfide Bridges in Peptides

DiPTH‐cystine as well as PTH‐cysteine have been recommended earlier for application under non‐reducing and reducing conditions, respectively, for the identification of disulfide connectivities in peptides and proteins as aforementioned [24, 28]. The recently reported application of Edman degradation for the detection of a disulfide bridge between Cys1 and Cys6 in oxytocin using the PPSQ‐50A protein sequencer system [23], however, remains unclear for the following reasons: (i) diPTH‐cystine from the second half‐cystine is found only in cycle 6 after several cycles in which the first half‐cystine can undergo significant degradation as already known [9, 24, 25, 26, 28]; (ii) only minor amounts were detected which might be considered insufficient, especially due to carry‐over effects from previous cycles and incomplete degradation that occurs in each sequencing cycle and may affect the background noise especially in late cycles of a sequencing run [25]; and (iii) the relevant peak is labeled as PTH‐cystine, which according to the sequencing data of the peptide, in which DTT was applied, cannot result in the major derivative and PTH‐cysteine most likely represents the corresponding compound here [24].

**FIGURE 2:**
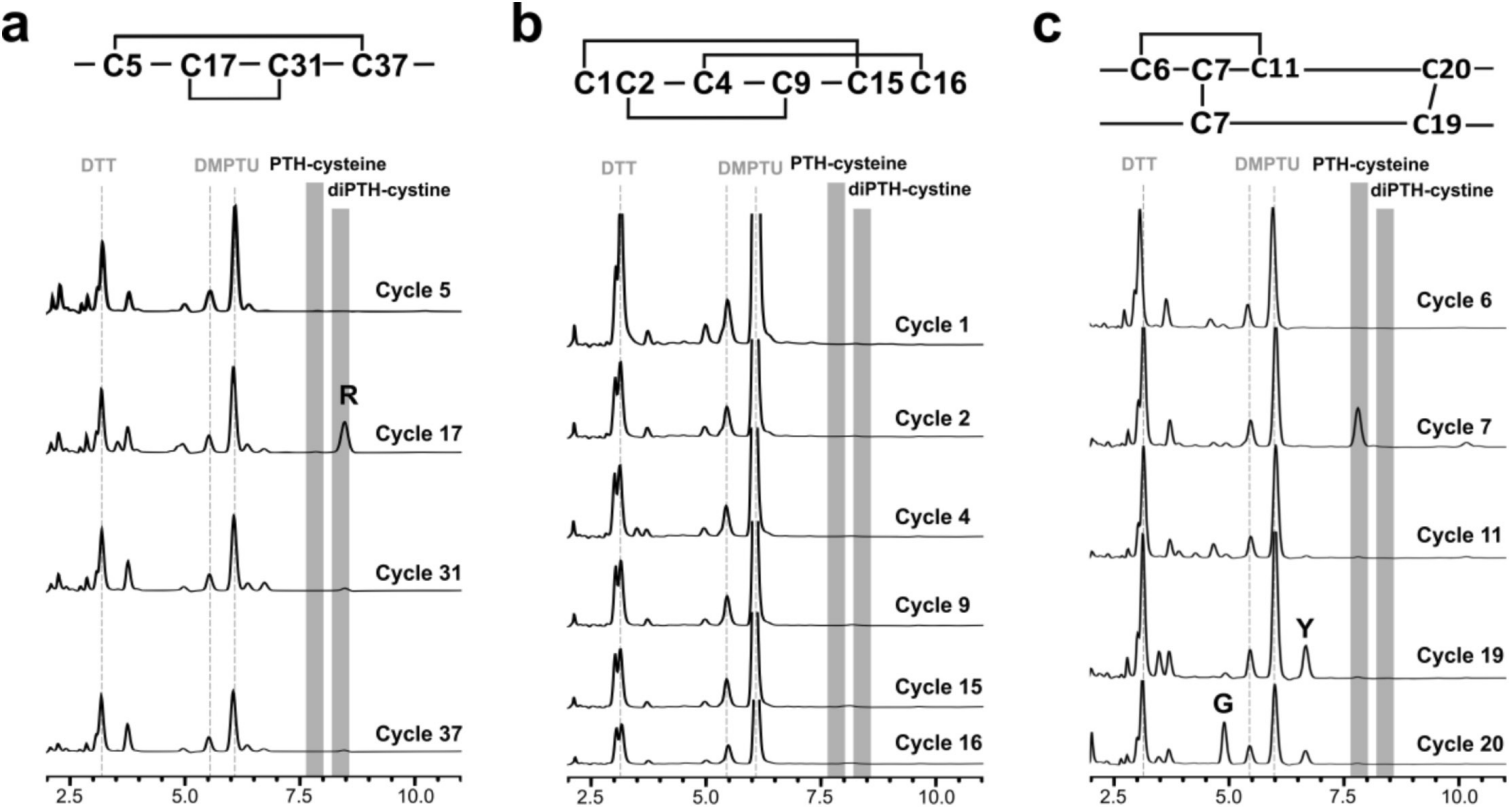
Chromatograms of cycles covering cysteine positions in (a) tridegin, (b) μ‐KIIIA, and (c) insulin. Frames in the chromatograms for Edman degradation indicate the retention times of PTH‐cysteine and diPTH‐cystine as indicated. Data of a, b, and c were reproduced from references [6] and [7]. DTT, dithiothreitol; DMPTU, (2,6‐dimethylphenyl)thiourea.

To identify and confirm the detected peak in the standard PPSQ protein sequencer system under reducing conditions (given by the manufacturer), we reviewed our previous analyses of three multiple disulfide‐containing peptides, namely human insulin, conotoxin μ‐KIIIA, and the tridegin analog B_[C19S,C25S]_ (Figure 2) [6, 7]. Tridegin is a 66mer peptide and the analog used for Edman sequencing contains two disulfide bridges between C5–C37 and C17–C31 (Figure 2a) [7]. The conotoxin μ‐KIIIA possesses three disulfide bridges with linkages between C1–C15, C2–C9, and C4–C16 (Figure 2b), whereas insulin consists of two peptide chains A and B that are linked by two intermolecular disulfide bridges between C7(A)–C7(B) and C20(A)–C19(B), a further intramolecular disulfide bridge is formed within chain A between C6(A)–C11(A) (Figure 2c) [6]. Considering the presence of the disulfide bridges within the sequences, it became apparent that in case of one disulfide bridge (C7(A)–C7(B), human insulin) both half‐cystines are cleaved in the same cycle. The cysteines of the other disulfide bridges are separated by a distance of at least four amino acids and, therefore, may already suffer from degradation of the first half‐cystine during the Edman degradation cycles [9, 24, 25, 26, 28]. In addition, in several cases they only occur in late cycles of a sequencing run. Therefore, in this study for analysis of diPTH‐cystine and PTH‐cysteine the complexity level of the peptides is limited to a maximum of three disulfide bridges and a maximum of two adjacent cysteines. It remains elusive how reliable more complex peptides, i.e., with more than three disulfide bridges or more than two adjacent cysteines, can be analyzed with respect to the identification of cysteines in Edman sequencing.

The most striking peak was present for the C7(A)–C7(B) disulfide bridge that was detected in the same cycle of Edman degradation of insulin (Figure 2c, cycle 7). Upon comparison of the retention times of our reference compounds for PTH‐cysteine and diPTH‐cystine (Figure 1), we identified the observed peak in the 7th cycle of human insulin as PTH‐cysteine (Figure 2c, cycle 7). This is in line with the fact that conversion of the diATZ‐cystine is performed in the presence of DTT, which will lead to reduction of the formed diPTH‐cystine to generate PTH‐cysteine [24, 28]. In addition, it even appeared advantageous to work under reducing conditions for the conversion reaction during Edman degradation since no overlap of the PTH‐cysteine was known for any of the other PTH‐amino acids in the PPSQ protein sequencer system (isocratic model). Interestingly, no PTH‐cysteine was detected when insulin was partially reduced and alkylated [6]. While insulin with 1 open bridge (2x carbamidomethylated, open bridge between C6(A) and C11(A)) still showed a peak for PTH‐cysteine, it disappeared completely in the case of partially reduced insulin with 2 open bridges (4x carbamidomethylated, further open bridge between C7(A) and C7(B)) [6]. This allowed for unambiguous annotation of the obtained peak as a cystine/disulfide bridge that must have emerged in the same cycle. For none of the other disulfide bridges, a peak for PTH‐cysteine or diPTH‐cystine was obtained, neither in the cycle of the first half‐cystine nor in the cycle of the second half‐cystine. Similar findings were described by others, i.e., detection of cystine with high recovery yields (20–30%) is most efficient when the second half‐cystine is present within the first 5–10 cycles [22, 26]. The peak was still known to be most intense when both half‐cystines were cleaved and converted in the same sequencing cycle [9, 22, 25, 27]. The reliability of this method remains elusive when cysteines are not involved in a disulfide bridge in the same cycle or when a disulfide bridge in the same cycle is measured late in sequencing (> 10 amino acids from the N‐terminus). So far, these options still appear to be less relevant for direct detection of disulfide bridges by Edman degradation without previous treatment by, e.g., partial reduction. Nevertheless, an appropriate design of a suitable sample work‐up procedure might significantly promote the applicability of this approach [25]. Especially when proteolytic digests are required, a convenient selection of enzymes and/or chemicals for protein digestion may be used to reinforce the formation of same cycle cysteines that could be found to form a disulfide bridge in the obtained fragments [25].

### 3.4 Annotation of Disulfide Bridges Utilizing Edman Degradation

**SCHEME 2:**
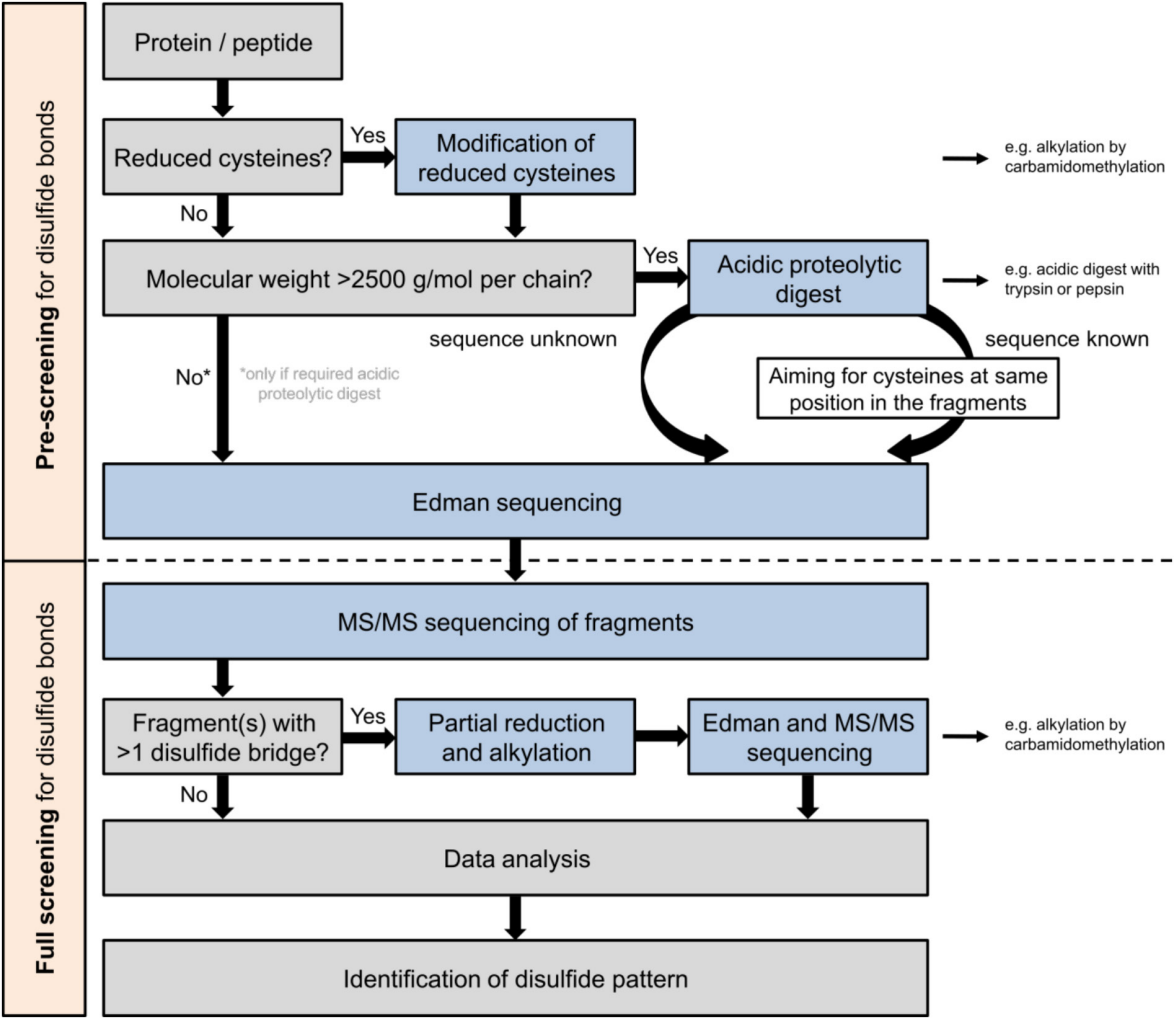
Workflow for the elucidation of the cysteine connectivity in peptides and proteins in a combined setup utilizing sequencing by Edman degradation and tandem mass spectrometry. Blue boxes indicate practical working steps, while gray boxes highlight decisions that need to be done or directly allow for data evaluation.

Edman degradation for the analysis of disulfide bridges is a promising application that should be re‐implemented in the bioanalytical tool box and further exploited for structure elucidation of purified peptides and proteins. However, neither mass spectrometry (as shown earlier [6]) nor Edman sequencing alone is sufficient for the analysis of all types of multiple disulfide‐bridged peptides and proteins [6], whereas a concerted utilization of both methods can significantly accelerate this process. Consequently, the workflow presented in Scheme 2 was developed comprising a pre‐screening step before a complete analysis of the disulfide bridges is performed if required. It should be noted that disulfide shuffling must be avoided at every step by appropriate adjustment of an acidic pH and/or modification of reduced cysteines prior to analysis. The obtained sample is subsequently analyzed by Edman sequencing applying suitable reference compounds for the cysteines, such as the recently re‐introduced PTH‐Cys (Cam) as an amino acid standard [6]. This workflow is of particular interest for peptides with a molecular weight of ≤ 2500 g/mol per peptide chain. In our experience, this molecular weight range yields sufficient intensities for PTH‐amino acids in the late cycles of Edman sequencing as well as suitable signal intensities for fragment ions in MS/MS sequencing. In addition, we recommend an acidic digest of the peptide or protein using a carefully selected protease or reagent that yields fragments containing cysteines at the same position of the respective sequence to identify disulfide bridges through detection of PTH‐cysteine of a high signal intensity during Edman degradation. Though analysis might also be performed by MS/MS sequencing, the ease of analysis and the reduced time expenditure for data evaluation are key arguments for the application of automated Edman sequencing, in particular in the pre‐screening process (Scheme 2). Based on the results obtained, peptides and protein fragments with disulfide bridges that could not be annotated up to that point are analyzed in a full screening approach as outlined by us earlier [6]. Specifically, re‐evaluation of these fragments containing one disulfide bridge by MS/MS sequencing should be performed to validate the corresponding connectivity. For peptides and fragments with more than one disulfide bridge, neither method easily reveals the corresponding pattern for the cysteine connectivity. Therefore, partial reduction and subsequent alkylation is required to obtain stepwise opening and derivatization of the disulfide bridges [6, 25]. These partially reduced and modified fragments can be analyzed again by applying either Edman or MS/MS sequencing to assign the missing disulfide bridges [6]. By combining each piece of information retrieved from the individual steps of the suggested workflow, a complete annotation of a disulfide‐bonded peptide or protein is possible. While this scheme will cover a broad range of proteins, its limitations in Edman sequencing, especially for the analysis of more complex peptides and proteins with more than three disulfide bridges and more than two adjacent cysteines, are still to be uncovered.

## 4 Conclusions

While there have been several approaches to applying Edman sequencing to elucidate disulfide bonds in peptides and proteins, much of this knowledge has remained increasingly unused since the advent of mass spectrometry for peptide and protein sequencing. However, it turned out that a thorough analysis using mass spectrometry alone is very time‐consuming. Furthermore, the structure of several proteins and peptides could not be clarified using this technique alone [6]. A combined approach utilizing the power of Edman sequencing and MS/MS techniques allows for a faster and easier analysis of the disulfide connectivity in complex multiply bridged peptides and proteins. This strategy also overcomes the challenges of mass spectrometry such as low signal intensities for fragment ions located directly adjacent to the cysteines that are involved in the disulfide bridges or the time‐consuming analyses carried out by well‐trained personnel.

For the first time, provision and appropriate characterization of diPTH‐cystine as well as PTH‐cysteine for the analysis by Edman sequencing was demonstrated in addition to their review as references in the current set‐up of the PPSQ sequencing device. These compounds represent valuable tools, especially in cases in which disulfide bridges present in the same cycle of a sequencing run, a situation which was already reported earlier to occur rather frequently in nature (e.g., in antibodies) or may be reinforced by appropriate choices of enzymes or reagents for fragmentation [25]. Their implementation as standards, including complete validation and application to a broader set of peptides and proteins, will be performed in the future.

Consequently, we introduce a workflow (Scheme 2) that allows scientists to quickly elucidate the disulfide connectivity of peptides and proteins using a combination of both techniques, in which Edman sequencing would be the starting point for immediate detection of disulfide bridges. A subsequent detailed analysis by mass spectrometry and Edman sequencing in a complementing fashion (potentially supported by partial reduction) would be significantly simplified if certain disulfide bridges were directly identified or excluded in advance.

## Author Contributions

D.I. and T.K. conceived the research, designed the experimental studies, wrote and reviewed the manuscript, and made available resources needed for the project; Y.E., A.T., and T.K. curated the data, wrote the original draft, and performed the formal analysis, investigation, and methodology in the lab. All authors discussed the results, contributed to, and approved the final manuscript.

## Conflicts of Interest

The authors declare no conflicts of interest.

## Supporting information


**FIGURE S1:** Analytical characterization by RP‐HPLC and mass spectrometry (inset) of diPTH‐cystine (gradient 20–60% acetonitrile containing 0.1% TFA [eluent B] in water containing 0.1% TFA [eluent A] in 40 min; calculated molecular weight 474.03 g/mol) (a) and PTH‐cysteine (gradient 10–50% eluent B in eluent A in 40 min; calculated molecular weight 238.02 g/mol) (b).

## Data Availability

The data that support the findings of this study are available from the corresponding author upon request.
