# Supplementary material for: DiPTH‐Cystine and PTH‐Cysteine in Disulfide Bond Analysis Using Automated Edman Degradation
Source: J Pept Sci. 2025 Aug 29;31(10):e70053. doi: 10.1002/psc.70053 (PMC12395892; doi:10.1002/psc.70053)
Supplement: Supplementary file 1 — FIGURE S1: Analytical characterization by RP‐HPLC and mass spectrometry (inset) of diPTH‐cystine (gradient 20–60% acetonitrile containing 0.1% TFA [eluent B] in water containing 0.1% TFA [eluent A] in 40 min; calculated molecular weight 474.03 g/mol) (a) and PTH‐cysteine (gradient 10–50% eluent B in eluent A in 40 min; calculated molecular weight 238.02 g/mol) (b). [file PSC-31-e70053-s001.docx]

**Supporting Information**

**DiPTH-cystine and PTH-cysteine in disulfide bond analysis using automated Edman degradation**

Toni Kühl^1,^*, Yomnah Y. Elsayed^2^, Alexander Terekhov^1^, Diana Imhof^1,^*

^1^Pharmaceutical Biochemistry and Bioanalytics, Pharmaceutical Institute, University of Bonn, 53121 Bonn, Germany

^2^Department of Pharmaceutical Analytical Chemistry, Faculty of Pharmacy, Ain Shams University, Organization of African Unity St. 11566 Cairo, Egypt


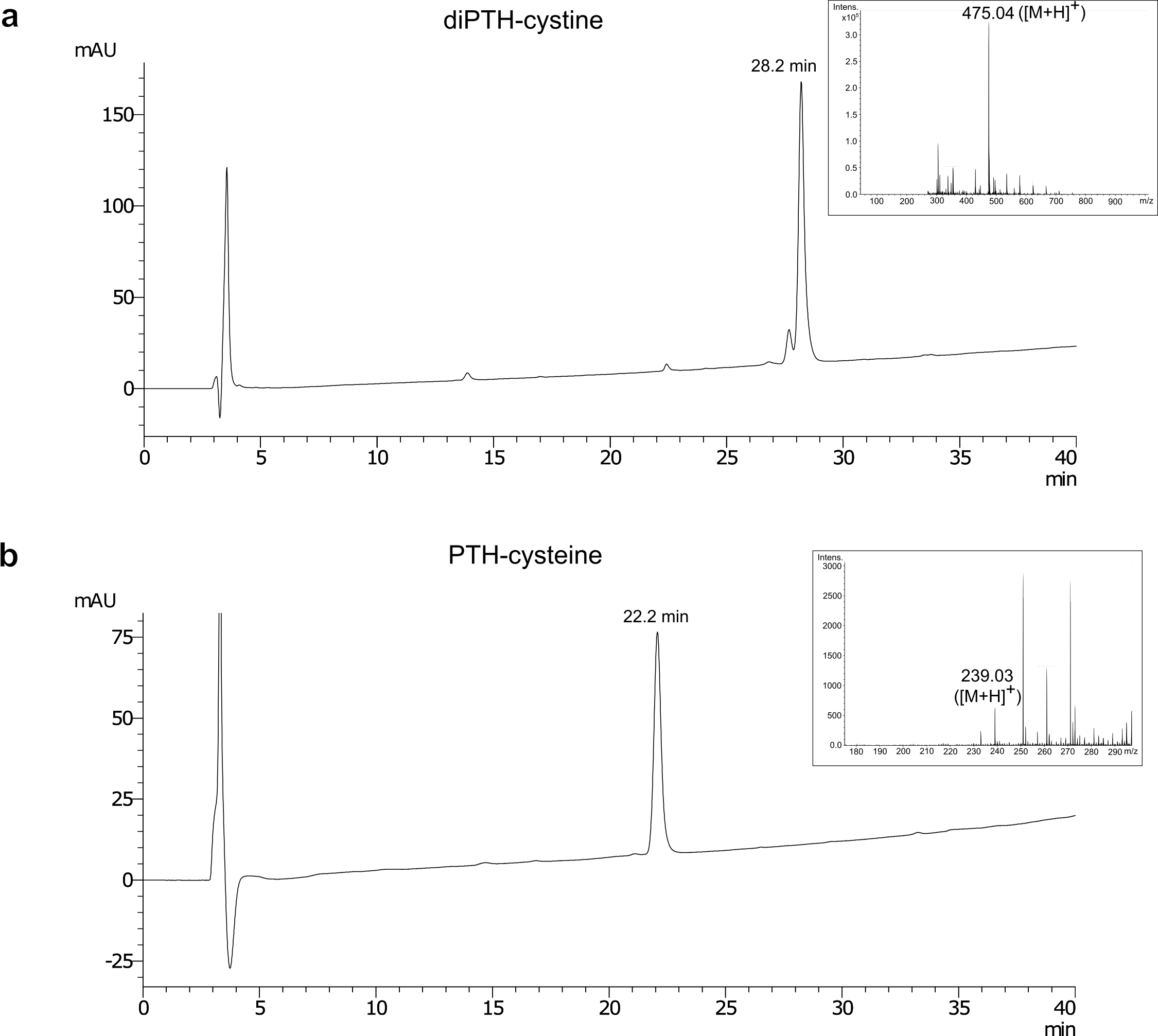


**Figure S1.** Analytical characterization by RP-HPLC and mass spectrometry (inset) of diPTH-cystine (gradient: 20-60% acetonitrile containing 0.1% TFA (eluent B) in water containing 0.1% TFA (eluent A) in 40 min; calculated molecular weight: 474.03 g/mol) (a) and PTH-cysteine (gradient: 10-50% eluent B in eluent A in 40 min; calculated molecular weight: 238.02 g/mol) (b).
